# Identification and genetic analysis of EMS-mutagenized wheat mutants conferring lesion-mimic premature aging

**DOI:** 10.1186/s12863-020-00891-x

**Published:** 2020-08-17

**Authors:** Weiwei Kong, Liming Wang, Pei Cao, Xingfeng Li, Jingjing Ji, Puhui Dong, Xuefang Yan, Chunping Wang, Honggang Wang, Jiaqiang Sun

**Affiliations:** 1 Agronomy College, Henan University of Science and Technology, Luoyang, 471023 Henan China; 2 Institute of Botany, Chinese Academy of Sciences, Beijing, 10093 China; 3 State Key Laboratory of Crop Biology/Agronomy College, Shandong Agricultrual University, Taian, 271018 Shandong China; 4 Institute of Crop Science, Chinese Academy of Agricultural Sciences, Beijing, 10081 China

**Keywords:** Wheat, *lmpa1*, Mutant, Chromosomal location

## Abstract

**Background:**

Lesion-mimic and premature aging (*lmpa*) mutant *lmpa1* was identified from the ethyl methane sulfonate (EMS) mutant library in the bread wheat variety Keda 527 (KD527) background. To reveal the genetic basis of *lmpa1* mutant, phenotypic observations and analyses of chlorophyll content and photosynthesis were carried out in *lmpa1*, KD527 and their F_1_ and F_2_ derivatives. Further, bulked segregation analysis (BSA) in combination with a 660 K SNP array were conducted on the F_2_ segregation population of *lmpa1*/Chinese spring (CS) to locate the *lmpa1* gene.

**Results:**

Most agronomic traits of *lmpa1* were similar to those of KD527 before lesion-like spots appeared. Genetic analysis indicated that the F_1_ plants from the crossing of *lmpa1* and KD527 exhibited the *lmpa* phenotype and the F_2_ progenies showed a segregation of normal (wild type, WT) and *lmpa*, with the ratios of *lmpa*: WT = 124:36(χ^2^ = 1.008 < =3.841), indicating that *lmpa* is a dominant mutation. The combination of BSA and the SNP array analysis of CS, *lmpa1* and *lmpa1*/CS F_2_ WT pool (50 plants) and *lmpa* pool (50 plants) showed that polymorphic SNPs were enriched on chromosome 5A, within a region of 30–40 Mb, indicating that the wheat premature aging gene *Lmpa1* was probably located on the short arm of chromosome 5A.

**Conclusions:**

EMS-mutagenized mutant *lmpa*1 deriving from elite wheat line KD527 conferred *lmpa*. *Lmpa* phenotype of *lmpa*1 mutant is controlled by a single dominant allele designated as *Lmpa1*, which affected wheat growth and development and reduced the thousand grain weight (*tgw*) of single plant in wheat. The gene *Lmpa1* was tentatively located within the region of 30–40 Mb near to the short arm of chromosome 5A.

## Background

Lesion-like mutants (*llm*) can spontaneously form spots on leaves, sheaths, or whole plants without significant damage, stress, or external pathogen infection [1]. The phenotype of *llm* is very similar to the hypersensitivity response (programmed cell death, PCD) after infection with pathogens [2]. Lesion-like spots (*lls*) formation is controlled by specific genes and/or affected by certain environmental conditions. They may be mostly caused by cell death and partially be correlated with pigment accumulation [3]. Previous researches [4] indicated that the mechanism of the lesion formation is very complicated because they may be controlled by genes related to disease resistance, regulation of death, and basic metabolic enzymes. Both signal molecules in plant defense to diseases and in environmental responses also play important role on the formation of *lls*.

In recent years, ethyl methane sulfonate (EMS) has been widely used to induce mutants with different agronomic traits in crops because it has the advantages of higher point mutation, fewer chromosomal aberrations, and easier screening of mutants over other methods [5–8]. EMS is a useful tool for improving particular agronomic traits, breeding new varieties, and screening elite germplasms [9]. Mutant germplasms induced by EMS can be effectively used to mine new genes, promote functional genomics studies, and accelerate breeding program [10].

To date, *llm* have been reported in corn [11], Arabidopsis [12], barley [13], and rice [14]. In recent years, wheat *lls* have been gradually found. For example, Geng [15] mapped a new wheat spot-like mutation gene *lm3*. Li et al. [16] found that wheat white spot mutation *I30* was controlled by a pair of recessive nuclear genes which were located on wheat chromosome 6D by using of BSA method and 660 K SNP array technology. Yao et al. [17] obtained a LLM from the crossing between normal parents Yanzhan 1 and Zaosui 30 and the LLM was controlled by two recessive genes named *lm1* and *lm2*.M66 [18], C591 (M8) [19], AIM9 [20], Ning7840 [21], HLP [22], LF2010 [23] and other wheat lesion mutants have also been reported.

Senescence is the final stage of plant development and an active process of extracting nutrients from old tissues. Premature aging can shorten the growth stage of crops, cause premature senility of functional organs earlier before grain filling [24], thus affecting crop yield and quality [25]. Many reports and in-depth studies on premature senescence in rice have been documented to date [26]. The gene of leaf premature senescence mutant *wss1* was located within 1200 kb near the centromere region of the long arm of chromosome 11 in rice [27]. Signs of senescence began to appear in the rice premature senescence mutant *es4* in about 60 days, due to the loss of function of the calcium-dependent protein kinase *OsCPK12* [28]. The 3-bp deletion in the gene of *WLS5* also leads to premature senescence in rice [29]. The early senescence mutations *esl2* [30], *esl3* [31], *esl4* [32], *esl5* [33], and *esl6* [34] selected by the Rice Research Institute of Southwest University by EMS mutation were controlled by mononuclear genes. Xiao et al. [35] located the mutant gene of premature aging mutant *zs* in the 600 kb region on the short arm of rice chromosome 12. The rice premature senescence gene *PLS2* was preliminarily determined to encode a glycosyltransferase by Wang et al. [36]. In recent years, wheat premature senescence has also been reported. Two additive QTLs on chromosomes 3A and 3B detected by Wei [37] were related to wheat early senescence indicators and six physiological traits related to premature senescence. An additive QTL controlling the flag leaf senescence was located between markers *gwm526* and *gwm382* on the long arm of chromosome 2A [38]. The leaf senescence gene *els1* was located on the chromosome of 2BS by bulked segregant RNA sequencing (BSR-Seq) method in common wheat [39]. T. Kajimura [40] used homologous cloning to obtain genes related to wheat aging, *TaSAG1-TaSAG9*, *TaSAG1*, *TaSAG3*, *TaSAG4*, and *TaSAG5* are genes related to amino acid metabolism, *TaSAG7* and *TaSAG8* are genes related to fatty acid metabolism, and *TaSAG9* is related to sugar and genes related to ribose metabolism, *TaSAG2* and *TaSAG6* are genes used to encode seed proteins.

This study reports the isolation of wheat lesion-like premature senescence mutants by EMS mutagenesis, and the genetic analysis of these mutants. The chromosomal localization of the premature aging gene was performed by the analysis of the segregating populations. This study generated germplasm resource for future cloning of new genes related to early senescence and exploring the regulatory mechanism of early senescence in wheat. It also laid a foundation for breeding new wheat varieties conferring resistance to premature senescence.

## Results

### Generation and identification of *lmpa* mutant

**Fig. 1:**
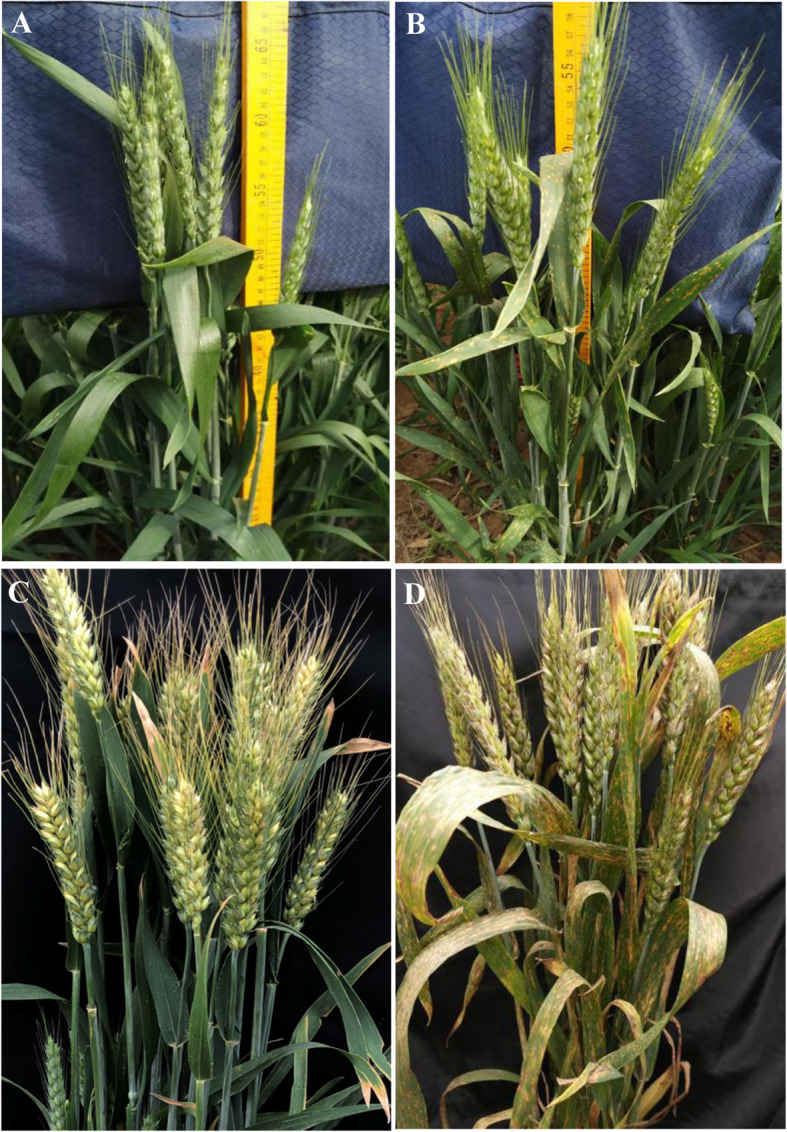
Phenotypes of the WT KD527 (**a**, **c**) and the mutants *lmpa1* (**b**, **d**) during late heading (**a**, **b**) and mid-late filling stage (**c**, **d**), respectively. Note: **a**: WT KD527 grows normally during late heading stage; **b**: A small amount of brown spots can be found on the leaves of mutant *lmpa1* at the late heading stage; **c**: KD527 grows normally at mid-late filling stage stage; **d**: *Lls* expand quickly to the leaves, stems and even spikes of *lmpa1*and premature aging appears during mid-late filling stage

**Fig. 2:**
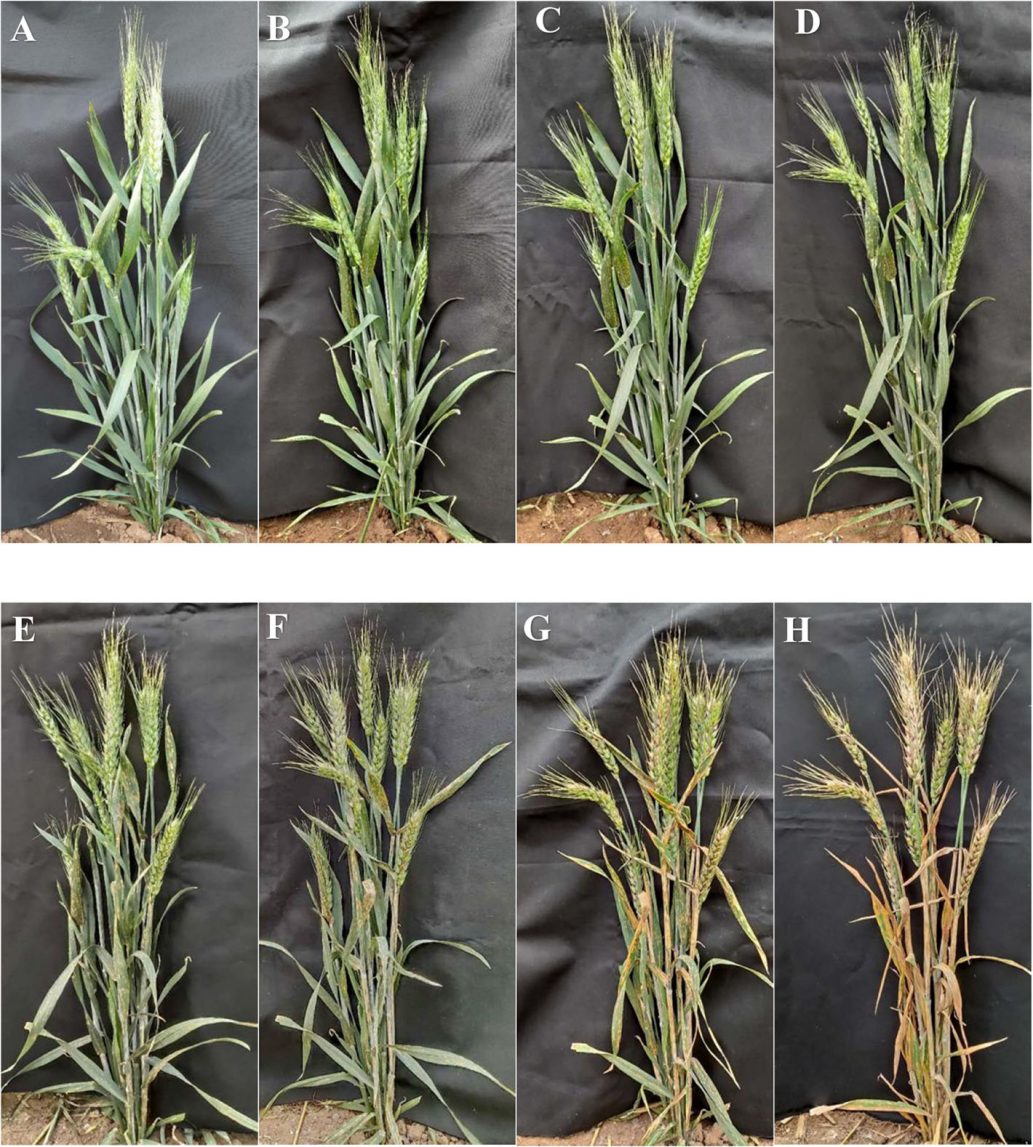
Formation, expansion and spread of *lmpa* of *lmpa1* mutant at different growth stages after heading in 2019. Note: **a**: *lmpa1* single plants have not been found *lls* on 23th April; **b**:*lmpa1* leaves have a small amount of brown spots on 24th April; **c**: *lmpa1* leaves have a significant increase in brown spots on 26th April; **d**: A large number of brown-yellow spots spread on the stem of *lmpa1* on 30th April; **e**: *lls* on the leaf spread to the leaf sheath, and a few brown-yellow spots appeared on the stem of *lmpa1* on 4th May; **f**:The brown and yellow spots on the stems continued to increase, and a few brown and yellow spots appeared on the spikes of *lmpa1* on 16th May; **g**: *lmpa1* leaves began to dry, and the brown and yellow spots on the spikes continued to increase on 20th May; **h**: *lmpa1* leaves and stems were withered, part spikes were drying up on 24th May

Multi-year agronomy comprehensive identification was used to select the *lmpa* mutant from the M_6_ generation induced by EMS in KD527 (Fig. 1a, c), which was named *lmpa1* (Fig. 1b, d). The agronomic identifications were conducted in KD527 and *lmpa1* and shown in Table 1. As can be seen from Table 1 and Fig. 1, the agronomic characters of *lmpa1* is similar to KD527 in plant height (*ph)*, flag leaf length (*fll*), ear length (*el*), number of grains per ear (*ngpe*) and other agronomic traits. The process of the formation of *lmpa* in *lmpa1* was observed throughout its whole growth period. *lmpa1* grows normally at seedling stage. After flag leaf picking, it appeared *lls* before senescence. Its leaves present some brown-yellow round disease spots which can gradually enlarge and expand. After heading, the disease spots quickly spread to the leaf sheath, stem and spike of *lmpa1*. With the extension of growth period, *lmpa1* emerges more and more disease spots over the whole plant and dried up even died during the filling stage (Fig. 2). In addition, the number of mutant individuals increases obviously after rain during grain filling and they get worse than before rain. The reason is unclear yet.

**Table 1:** Statistical analysis of *lmpa* traits in mutants and their hybrid progenies

Material / Combination	Generations	Phenotype			Separation ratio	Theoretical ratio	Χ^2^	*P*
		*lmpa*	WT	Total				
KD527		/	WT	All	/	/	/	/
*lmpa1*		*lmpa*	/	All	/	/	/	/
*lmpa1*/KD527	F_1_	10	0	10	/	/		
	F_2_	124	36	160	3.44	120:40	1.008	0.465

Note: df = 1; $$ {\upchi}_{0.05}^2 $$ =3.841, $$ {\upchi}_{0.01}^2 $$ =6.635

### Genetic analysis of *lmpa* mutant

In order to clarify the inheritance and genetic effects of the *lmpa* traits, the single plant *lmpa* traits and other agronomic traits of *lmpa1*, KD527 and CS crosses F_1_ and F_2_ were investigated. The results were statistically shown in Tables 1 and 2.

**Table 2:** Agronomic traits of KD527, *lmpa1* and their hybrids

Materials		ph (cm)	fll (cm)	el (cm)	ngpe	tgw (g)	ypp (g)
KD527		58.3 ± 2.9 ^a^	17.4 ± 0.4 ^a^	9.7 ± 0.5 ^a^	62.7 ^a^	54.45 ^a^	27.31 ^a^
*lmpa1*		50.3 ± 3.3 ^c^	17.2 ± 0.6 ^ab^	8.8 ± 0.6 ^c^	52.5 ^c^	42.17 ^c^	17.71 ^c^
*lmpa1*/KD527	F_1_	55.9 ± 4.5 ^ab^	16.8 ± 0.7 ^b^	9.5 ± 0.5 ^ab^	57 ^b^	44.67 ^bc^	20.37 ^bc^
	F_2_ *lmpa*	52.6 ± 2.1 ^bc^	16 ± 0.5 c	9.1 ± 0.7 ^bc^	57.51 ^b^	43.50 ^c^	20.01 ^bc^
	F_2_ WT	54.6 ± 2.2 ^b^	16.6 ± 0.2 ^b^	9.4 ± 0.4 ^ab^	59.3 ^ab^	49.17 ^b^	23.33 ^b^

Note:^a,b,c^ significant difference at 0.05 level

**Fig. 3:**
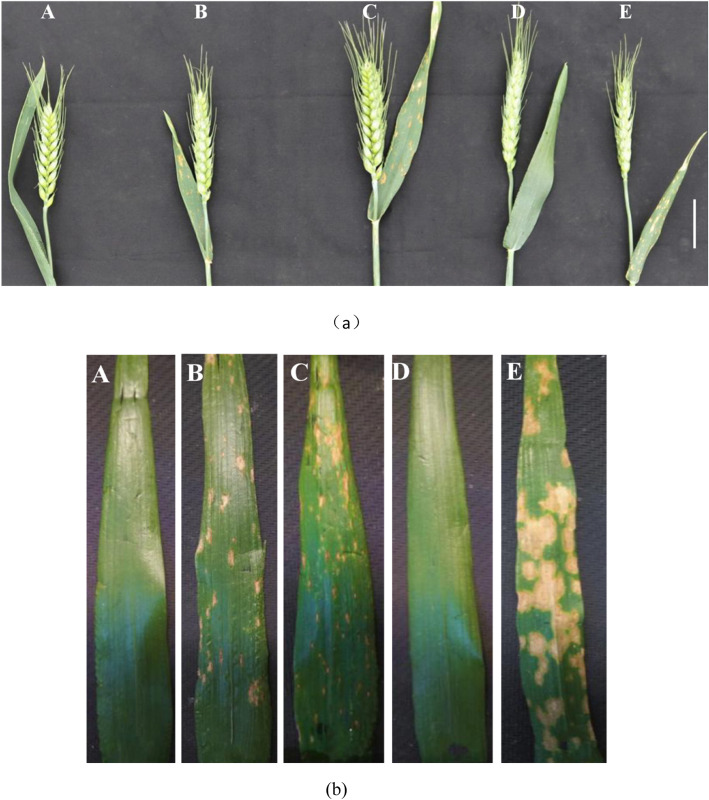
Phenotype of spikes (**a**) and flag leaves (**b**) of KD527(**a**) and *lmpa1*(**e**) and the F_1_ (**b**) and F_2_ (**c**, **d**) offspring of *lmpa1*/KD527 (bar = 2 cm). Note :**a**: KD527, WT; **b**: *lmpa*1/KD527 F_1_, *lmpa*; **c**: *lmpa*1/KD527 F_2_ *lmpa*; **d**: *lmpa*1/KD527 F_2_, WT; **e**: *lmpa*1, *lmpa*

As can be seen from Table 1 and Fig. 3, the KD527 plants behaved normally, *lmpa1* suffered from plaque-like premature aging. The plants of the constructed hybrid F_1_ population all showed lesion-like premature senescence characters. The plants from the F_2_ population showed two types of premature senescence plants and normal plants. Chi-square test showed that *Lmpa1* gene is dominant and conforms to the separation ratio of single gene 3:1.

*lmpa1* had shorter *el* and lower *npge* than that of KD527 (Table 2). The thousand grain weight (*tgw)* and yield per plant (*ypp)* in F_2_ were significantly higher than those in *lmpa* plants, indicating that the mutant’s early-like traits could significantly reduce wheat yield. However, the reduction extent to which it causes wheat yield and whether it has other disease resistance still needs further identification.

### Photosynthetic assay of *lmpa* mutants

**Fig. 4:**
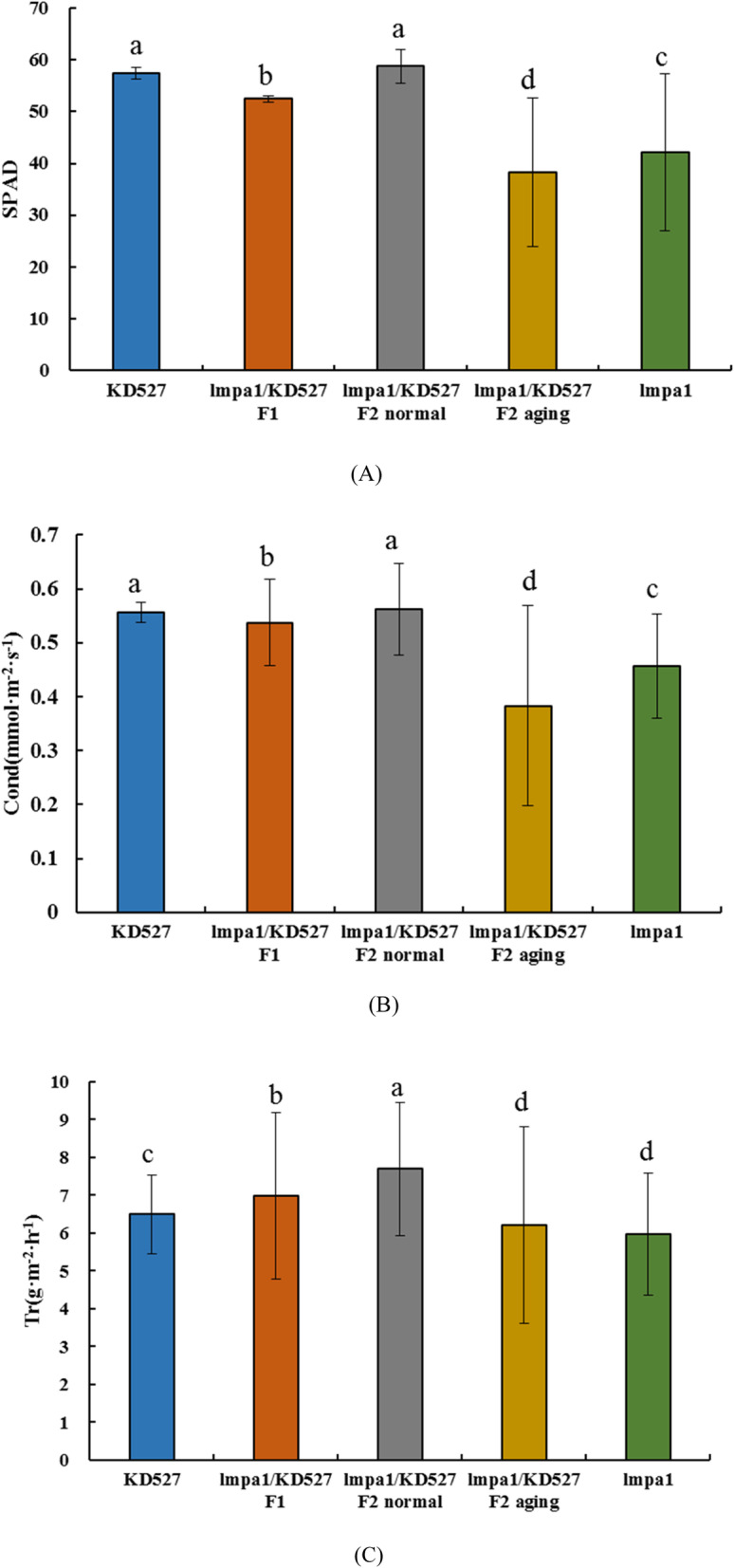
Determination of photosynthetic physiological indexes of KD527 and *lmpa1* and their hybrids. **Note**:**a**: relative chlorophyll content (SPAD); **b**: stomatal conductance (mmol·m^− 2^·s^− 1^); **c**: transpiration rate (g·m^− 2^·h^− 1^); **a**,**b**,**c**: significant difference at 0.05 level

In order to further understand the effects of mutants on wheat photosynthetic physiology, *SPAD-502 Plus* and *LI-6400 XT* were used to measure chlorophyll content (SPAD), stomatal conductance (Cond), and transpiration rate (Tr) of KD527, *lmpa1*, and their hybrids in the field (Fig. 4).

Physiological indicators comprising SPAD, Cond and Tr of *lmpa1*/KD527 F_1_ were higher than that of *lmpa1*. However, these indicators in normal plants from F_2_ population were not significantly different from that of KD527 and were significantly higher than those of *lmpa* plants. It indicated that the *lmpa* mutant had a significant effect on wheat photosynthetic physiological process. As a result, *lmpa1* affected wheat growth and development so seriously that the plant cannot age normally and premature senescence occurs, which may also be one of the reasons for reducing the thousand grain weight of single plant in wheat.

### Chromosomal location of *Lmpa1* gene

**Fig. 5:**
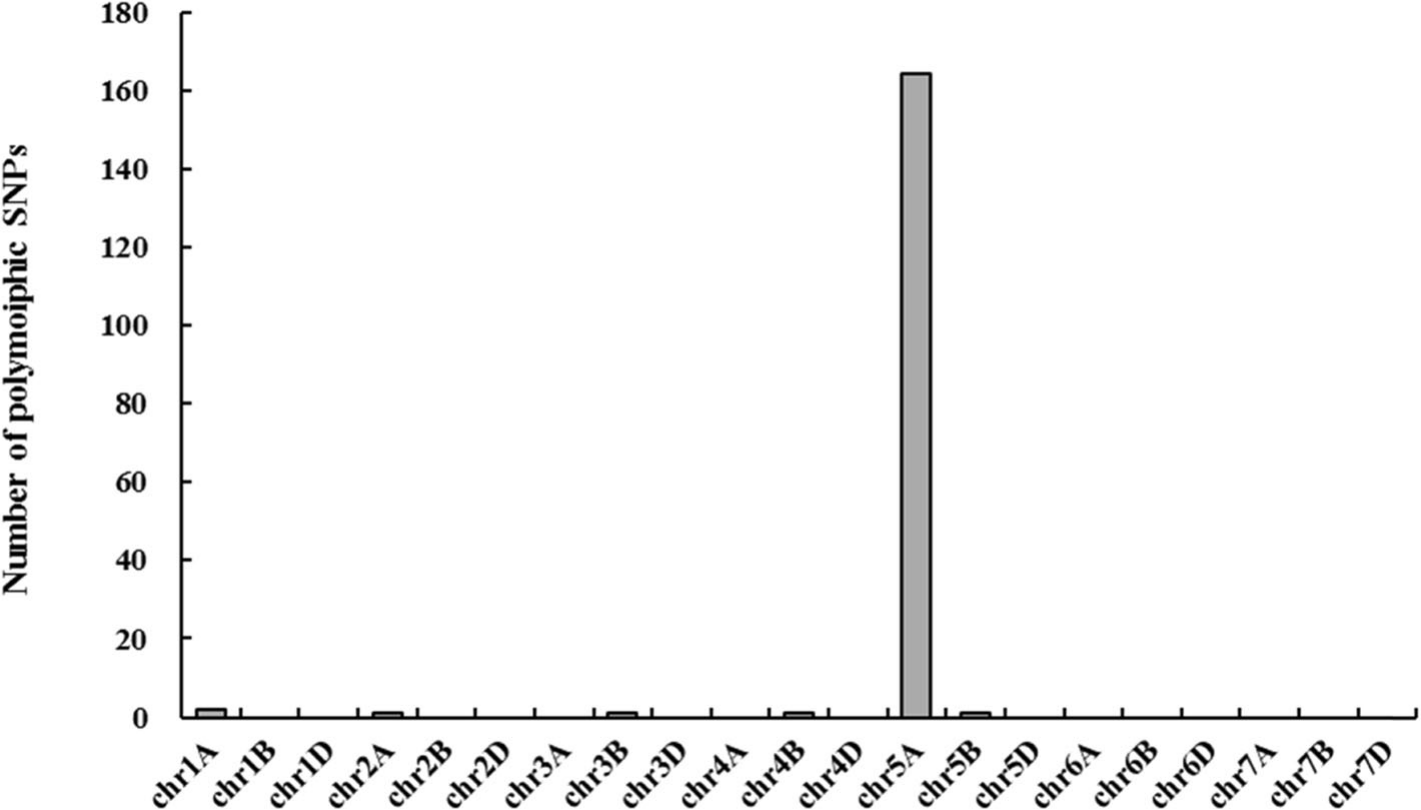
Distribution of polymorphic SNPs on each chromosome

**Fig. 6:**
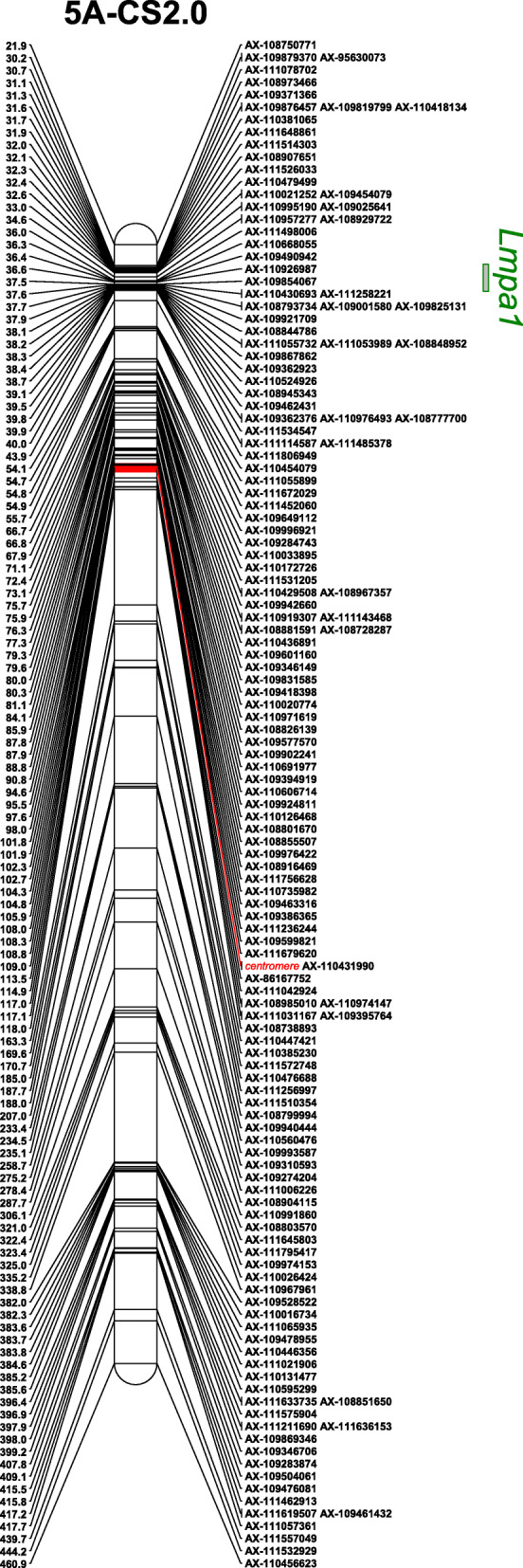
Genetic linkage map of SNPs related to premature aging gene on chromosome 5A. Notes: The red segment indicates the estimated centromeric region. The rectange in green on the right of the chromosome indicates the estimated chromosomal region of the gene *Lmpa1*

DNA samples from CS, *lmpa1*, and mixed samples of normal plants (50) and premature senescent plants (50) in the F_2_ population of combination *lmpa1*/CS was used to construct a BSA pool for 660 K SNP array analysis. As a result, 170 polymorphic SNP loci distributed on chromosomes 1A, 2A, 3B, 4B, 5A, 5B respectively were found (Fig. 5) and 164 SNP loci were located on chromosome 5A. It is presumed that the *Lmpa1* gene is located on the 5A chromosome of wheat. Based on physical positions of the polymorphic SNPs in Chinese Spring (IWGSCv2.0), a genetic linkage map of SNPs linked with *Lmpa1* genes on chromosome 5A were constructed by MapChart (Fig. 6). The results showed that most of the polymorphic SNPs are enriched within a 30-40 Mb region near to the short arm of chromosome 5A, indicating that the *LMPA1* gene is highly possible within this region.

### Screening of candidate genes related to *Lmpa1*

Based on the results of the 660 K SNP chip, we used the website of JBrowse (http://202.194.139.32/jbrowse-1.12.3-release/?data = Chinese_Spring) to screen related genes in the 30-40 Mb segment of the short arm of wheat 5A chromosome. A total of 120 genes were found within the 30-40 Mb region of chromosome 5A. And 13 genes related to plant growth and development may be the candidate genes associated with *Lmpa1* (Table 3).

**Table 3:** List of Candidate Genes Related to Wheat Early Aging

Gene name	Gene annotation	Gene length (bp)	Protein length (aa)
*TraesCS5A01G034300.1*	Protein kinase superfamily protein	1194	397
*TraesCS5A01G035200.1*	Protein kinase family proteins	2346	781
*TraesCS5A01G037100.1*	Kinase family proteins	1831	477
*TraesCS5A01G043600.1*	Protein kinase family proteins	2148	715
*TraesCS5A01G038300.2*	Auxin response factor	4008	899
*TraesCS5A01G039100.1*	peroxidase	1250	340
*TraesCS5A01G039400.1*	peroxidase	1129	277
*TraesCS5A01G039500.1*	peroxidase	1400	340
*TraesCS5A01G040300.1*	Zinc finger protein	1246	287
*TraesCS5A01G040500.1*	Remorin	1800	449
*TraesCS5A01G041500.1*	Myb-related transcription factor	390	309
*TraesCS5A01G041600.2*	Pentapeptide repeat superfamily protein	1248	266
*TraesCS5A01G042500.1*	Protein TRIGALACTOSYLDIACYLGLYCEROL 2	1660	446

## Discussions

Premature senescence is a phenomenon that aging of plants physiological and biochemical process in their growth period takes place earlier than that of normal plants. Premature senescence in cereal crops such as wheat, rice and corn, will affect the production of photosynthetic products and their transportation and accumulation into grains and in turn decrease grain yield. Premature aging mutants can be regarded as an important tool to understand premature senescence and benefit elucidating the PCD in plants. Precious researches on premature senescence mainly focus on rice premature senescence mutants and their gene mapping. There are few reports on the creation of wheat premature senescence mutants, the main ones are some types of leaf premature aging. For example, M.M.Li [39] developed 7 polymorphic markers linked to the early leaf senescence gene els1 by applying a large number of segregant analyses and RNA-Seq.

This study reports the *lmpa1* mutant deriving from the EMS-induced mutant library in the KD527 background. The mutant with the characteristics of both lesion-like spots and premature senescence, will enrich the wheat premature senescence mutant library and lay the germplasm foundation for further research on the traits related to early senescence in wheat.

In this study, we characterized the mutants *lmpa1* and analyzed its photosynthetic physiology. We found that lesion-like spots and premature senescence can significantly affect *el*, seed setting rate (*ssr*), *tgw* and other agronomic traits in wheat. They can reduce the expression of chlorophyll, cause the physiological dysfunction of leaves and decrease the ability of photosynthetic assimilation. As a result, the grain filling time was shortened, the dry matter accumulation of the grain was reduced, the *ssr* and the *tgw* were affected, and the yield and quality were damaged. In order to better understand the physiological and biochemical mechanisms of premature senescence, we will refer to B.F.Wang’s [41] methods on premature aging mutants in rice. It is planned to use cell histochemical staining, determination of net photosynthetic rate and photosynthetic pigment content and determination of enzyme activity to find physiological and biochemical indicators related to senescence. In the meantime, cell morphology of mutants will be observed by transmission electron microscope. And the expression of gene related to senescence and hormone content in mutants will be analyzed. The differences in physiological and biochemical, hormone, and cell morphology between premature aging mutants and normal plants will be discussed. It has been reported that rice lesions-like mutant *spl41* [42] can enhance resistance to rice bacterial leaf blight. Therefore, disease resistance of *lmpa1* should be identified in the future.

In this study, the *Lmpa1* was located within 30–40 Mb region on chromosome 5A by using of SNP chip sequencing and BSA analysis. Up to date, there is no report of premature aging gene on the chromosome 5A in wheat. Among the 13 candidate genes, the candidate gene *TraesCS5A01G040300.1* encoding a zinc finger protein is similar to the zinc finger transcription factor found in wheat leaf premature senescence mutant *m68* [43] and may be associated with premature aging. It is important to screen premature aging genes and explore the causes and mechanisms of premature aging. It was found [44] that water deficit during grain filling period could cause premature senescence of flag leaves, but the senescence process could be delayed by changing hormone concentration of plants. There are many reasons for rice premature aging, for example, the effect of NAC transcription factors on abscisic acid (ABA) [25], the functional impairment of calcium-dependent protein kinase *OsCPK12* [28], the deletion of gene fragment [29], the response and regulation of genes related to antioxidant and carbohydrate metabolism [45], and so on. Based on the research experience of rice early senescence, further work should be focused on the cloning and functional verification of candidate gene for premature aging. The effects of premature aging on protein expression, hormone signaling pathways, and gene expressing related to metabolism, will be emphasized in order to further reveal the molecular mechanism of wheat premature senility.

## Conclusions

We identified an EMS-mutagenized mutant *lmpa1*, which derived from elite wheat line KD527 and conferred *lmpa*. Genetic analysis indicated that the *lmpa* phenotype of *lmpa1* mutant is controlled by a single dominant allele designated as *Lmpa1*, which affected wheat growth and development and reduced the *tgw* of single plant in wheat. By applying BSA method and 660 K SNP Chip sequencing, the gene *Lmpa1* was tentatively located within the region of 30–40 Mb near to the short arm of chromosome 5A.

## Methods and materials

### Plant materials

The materials for this study were the bred new wheat line KD527 (from our laboratory), the *lmpa1* mutant (isolated from the EMS mutant library of KD527), the F_1_ and F_2_ hybrid populations of *lmpa1* and KD527, and *lmpa1* and F_1_ and F_2_ populations crossed by CS. All materials from the Wheat Germplasm Innovation Group of the Henan University of Science and Technology are maintained and planted in Luoyang City, Henan Province, China.

### EMS mutagenesis

Seeds were soaked with distilled, deionized water at room temperature for 16 ~ 20 h until seeds completely absorb water and fully swell. Seeds were then treated with 0.3% EMS in phosphate buffer, pH 7 at room temperature for 4 ~ 6 h. The treated seeds were then rinsed in tap water for 12 h, dried for 30mins, and immediately sown in the field.

### Screening of *lmpa* mutants

EMS-mutagenized KD527 seeds were grown with row spacing of 20 cm and plant spacing of 5 cm. Individual plants with lesion-like spots were identified from the M_0_ population materials and harvested as M_1_. In the second year, M_1_ seeds were grown in the field, evaluated for their agronomic traits during growth, and harvested as a single plant as M_2_. M_2_ were planted and evaluated on the stability of mutant traits during their growth. From M_3_ generation on, field investigation was conducted every 7 days. Ten plants were randomly selected from typical mutant lines. The plant height (*ph*), plant spike number (*ppn*), panicle length (*pl*), panicle grain number (*pgn*) and other agronomic traits were investigated and evaluated. All stable mutant individuals were harvested and threshed to survey ear length (*el*), thousand-grain weight (*tgw*), yield per plant (*ypp*) and other seed traits. The agronomy identification and stability evaluation were carried out continuously in M_4_ and M_5_ generations. Finally, the stable mutant *lmpa1* was bred in M_6_ generation.

### Construction of segregating population of premature senescence mutants

*lmpa1* was first crossed with KD527. The F_1_ seeds were harvested on a single plant basis and were planted in the field to investigate the lesion-like spot premature senescence trait and other agronomic traits during the growing period. They were harvested as F_2_ seeds. The lesion-like spot premature senescence trait and other agronomic traits of the individual plants in F_2_ population were also investigated to determine the genetic mode and genetic effect of the *LMPA* gene.

*lmpa1* was also crossed with CS as described above. Based on the lesion-like spot premature senescence trait of the F_2_ population, the leaf DNA samples of typical *Lmpa* and normal individual plants were extracted and combined as a BSA pool for the 660 K SNP array sequencing analysis to locate *LMPA* gene to specific chromosome. DNA extraction from leaves was performed according to the methods described by Y. Wang et al. [46].

### Measurement of chlorophyll content and photosynthesis activities

After wheat heading, the chlorophyll content and photosynthesis activities of *lmpa1,* KD527 and their hybrid F_1_ and F_2_ populations were measured by using chlorophyll meter *SPAD-502Plu* (Konica Minolta, Japan) and portable photosynthesis meter *LI-6400 XT* (LI-COR, American) on May 1st and May 18th, 2019, respectively. The measurement methods were strictly in accordance with the operation manuals.

### Chromosomal location analysis

Fifty typical *lmpa* plants and 50 normal plants selected from the *lmpa1* x CS F_2_ population were combined respectively into two DNA BSA pools marked *lmpa* pool (LP) and normal pool (WT). The 660 K SNP chip analysis was conducted by Zhongyujin Label Biotechnology Co., Ltd. in Beijing, China. Using the DNA pools of CS and *lmpa1* as controls, the candidate chromosome segments were estimated by screening significant differences in allele frequencies (AF) of polymorphic sites (SNPs) between the two BSA pools of LP and WT respectively.

## Data Availability

The data sets supporting the results of this article are included in this manuscript. The datasets generated and/or analysed during the current study are available in the http://plants.ensembl.org/Triticum_aestivum/Tools/Blast?db=core.

## Abbreviations

*Lmpa*: Lesion-mimic and premature aging
EMS: Ethyl methane sulfonate
KD527: Keda 527
BSA: Bulked segregation analysis
CS: Chinese spring
WT: Wild type
*tgw*: Thousand grain weight
*llm*: Lesion-like mutants
PCD: Programmed cell death
BSR-Seq: Bulked segregant RNA sequencing
*ph*: Plant height
*fll*: Flag leaf length
*el*: Ear length
*ngpe*: Number of grains per ear
*ypp*: Yield per plant
Cond: Stomatal conductance
Tr: Transpiration rate
*ssr*: Seed setting rate
ABA: Abscisic acid
*ppn*: Plant spike number
*pl*: Panicle length
*pgn*: Panicle grain number
LP: *Lmpa* pool
AF: Allele frequencies
SNPs: Single nucleotide polymorphism sites
